# Evolving Perceptions and Attitudes to Adopting Generative AI in Professional Settings: Multicenter Longitudinal Qualitative Study of Senior Chinese Hospital Leaders

**DOI:** 10.2196/75531

**Published:** 2025-06-27

**Authors:** Zheng Zhi, Jing Zhao, Qiang Li, Qingxia Li, Meifang Xu, Yan Zuo, Ming Wang, Jiankang Liu, Jingyi Guan, Jia Wang

**Affiliations:** 1 Hebei University of Chinese Medicine, Shijiazhuang, China; 2 Hebei Province Traditional Chinese Medicine Hospital, Shijiazhuang, China; 3 Department of Oncology, Hebei General Hospital, #348 Heping West Road, Xinhua District, Shijiazhuang, 050051, China, 86 13653115081; 4 Hebei General Hospital, Shijiazhuang, China; 5 General Office, Shulan (Hang Zhou) Hospital, Hangzhou, China; 6 Department of Gynecology and Obstetrics Nursing, West China Second University Hospital, Chengdu, China; 7 West China School of Nursing, Sichuan University, Chengdu, China; 8 Key laboratory of Birth Defects and Related Disease of Women and Children (Sichuan University), Ministry of Education, Chengdu, China; 9 Department of Oncology, Weihai Central Hospital Affiliated to Qingdao University, Weihai, China; 10 Graduate College, Hebei University of Chinese Medicine, Shijiazhuang, China

**Keywords:** attitude, generative artificial intelligence, GenAI, longitudinal study, perception, qualitative study, senior hospital leader, technology adoption

## Abstract

**Background:**

The rapid evolution of generative artificial intelligence (GenAI) is transforming health care globally. In China, hospitals are rapidly embracing digital transformation. Senior leaders are pivotal in influencing and deciding the adoption of GenAI technologies in professional settings. However, evidence on their perceptions and attitudes and how they evolve over time is lacking.

**Objective:**

This study aims to investigate senior Chinese hospital leaders’ perceptions relating to GenAI and their attitudes toward adopting GenAI technologies in professional settings and to document how their perspectives evolve.

**Methods:**

A longitudinal, descriptive qualitative study was conducted using a phenomenological approach. Senior leaders, including hospital directors/deputies and department heads/deputies, from 3 tertiary hospitals across the Hebei, Guangdong, and Sichuan provinces were purposively sampled. Data were collected through semistructured telephone interviews at two time points (baseline and 6 months later). Interview transcriptions were analyzed using Colaizzi’s method to extract themes related to knowledge acquisition, attitudinal shifts, and evolving barriers and facilitators to GenAI adoption.

**Results:**

A total of 32 participants were interviewed in August 2024 and February 2025, including 11 (34.4%) participants from Hebei, 12 (37.5%) from Sichuan, and 9 (28.1%) from Guangdong. Their roles included 8 (25%) hospital directors/deputies and 24 (75%) department heads/deputies. The analysis of the interview transcriptions revealed three primary themes. First, participants’ understanding of GenAI improved markedly over time as they engaged with diverse information sources and gained practical experience. Second, despite widespread support for personal use, senior leaders shifted from initial reluctance to formal institutional adoption. Third, initial obstacles, such as limited technical literacy and resource constraint concerns, diminished over time, while new drivers, including peer influence and technological breakthroughs such as DeepSeek, emerged to catalyze adoption.

**Conclusions:**

Senior Chinese hospital leaders’ perceptions and attitudes toward GenAI have evolved significantly over time. Improved knowledge through diverse information channels has enhanced their comprehension and shifted their stance from cautious personal experimentation toward formal institutional adoption. The dynamic interplay between diminishing obstacles and emerging catalysts, notably the influence of peer practices and technological breakthroughs such as DeepSeek, underscores the potential for transformative change in health care management. Targeted educational initiatives, pilot projects, and robust policy frameworks are needed to facilitate GenAI integration.

## Introduction

The rapid evolution of generative artificial intelligence (GenAI) technologies is reshaping industries fundamentally, including health care [1-3]. GenAI offers unprecedented capabilities in natural language processing, image synthesis, and data analysis, which can streamline clinical decision-making, optimize administrative processes, and ultimately enhance patient care [3,4]. In the context of Chinese hospitals, where rapid digital transformation is actively encouraged by government policies and market competition, the adoption of GenAI represents a strategic opportunity. Senior leaders are increasingly tasked with integrating the technologies to address complex challenges such as resource constraints, operational inefficiencies, and the need for improved diagnostic accuracy [4,5].

The adoption of GenAI in health care is a rapidly evolving field with significant potential and challenges. GenAI technologies, such as generative adversarial networks and large language models, are being integrated into various health care domains, including medical imaging, drug design, and mental health care, offering transformative possibilities [6,7]. However, the deployment of GenAI in health care is not without hurdles. Ethical and regulatory challenges, particularly concerning data privacy and security, are prominent, necessitating stringent guidelines and continuous oversight [8,9]. The integration of GenAI also requires a cultural shift within health care systems to maximize its value, emphasizing a bottom-up adoption approach to enhance patient outcomes [10]. Despite the challenges, GenAI holds promise for improving health care quality and operational efficiency, provided that institutions address governance, workforce training, and ethical oversight [11,12]. The health care sector’s readiness to implement complementary innovations alongside GenAI suggests a potential for more rapid improvements compared to past technologies [13]. Notably, our study coincided with DeepSeek’s launch in China and its global debut. Its release is a catalyst for rapid GenAI uptake, which suggests that a national-level endorsement and a major technical breakthrough accelerated both awareness and institutional interest.

Despite an expanding literature on GenAI, few studies have examined the perceptions and attitudes of senior hospital leaders, who are key decision makers in technology adoption, and how their perspectives evolve in a rapidly changing technological landscape. Existing research predominantly addresses the technical and operational aspects of GenAI implementation, rather than exploring the leadership dimensions that drive adoption decisions and outcomes. Consequently, there is a notable gap in evidence capturing how senior hospital leaders perceive emerging GenAI technologies and how their attitudes toward adopting these innovations in professional settings evolve.

This study addressed the gap by conducting a 6-month longitudinal qualitative investigation into the evolving perceptions and attitudes of senior leaders across multiple Chinese hospitals. Our primary objectives were to investigate their perceptions relating to GenAI and their attitudes toward adopting GenAI technologies in professional settings, and document how their perspectives evolve over time as they interact with this emerging technology. In this study, we define perception as the combination of awareness of GenAI’s existence and accurate knowledge of its core functions.

By focusing on the voices of senior leaders, our findings may advance our theoretical understanding of leadership in technology adoption and provide practical insights for policy makers and hospital administrators aiming to foster digital transformation in health care. By capturing the progression from initial uncertainty to informed optimism, our results stress the role of continuous education, experiential learning, and strategic pilot projects in overcoming barriers to GenAI integration, and offer evidence for designing targeted leadership initiatives to support sustainable innovation and adoption in rapidly evolving health care environments and informing future quantitative research.

## Methods

### Study Design

This longitudinal descriptive qualitative study used a phenomenological approach to systematically track changes in senior leaders’ perspectives on GenAI adoption over 6 months [14]. The study design incorporated two waves of data collection (August 2024 and February 2025) to capture evolving awareness and attitudes. The interval was selected to allow sufficient time for changes while minimizing recall bias. The phenomenological approach was chosen to prioritize participants’ lived experiences of navigating GenAI adoption in their professional roles, which aligned with established methodologies for studying technology assimilation in health care leadership contexts [15].

### Participant Recruitment

Participants were purposively selected through the professional network of the first author (ZZ) from three tertiary hospitals located in Hebei, Guangdong, and Sichuan provinces to represent regional diversity in China’s health care infrastructure [16,17]. The sample comprised senior leaders holding executive positions with direct authority over institutional technology decisions, including hospital directors/deputies and department heads/deputies responsible for digital technology adoption initiatives. Participants were contacted by phone and informed of the purpose and process of the study and the rights of participants before being invited. The first interviews were conducted immediately or during a scheduled appointment, at the end of which an appointment was made for the second interview. Recruitment continued until theoretical saturation was achieved, with two consecutive interviews yielding no novel analytic information [18,19]. All interviews in both waves were conducted with the same participant cohort. Notably, we enrolled traditional Chinese medicine (TCM) hospital leaders to enrich sample diversity and ensure our findings apply beyond Western medicine contexts.

### Data Collection

Data were collected through semistructured telephone interviews guided by a protocol organized into three investigative domains [20,21]. The first domain focused on overall perceptions of GenAI, probing participants’ awareness (“Have you heard of GenAI?”) and self-assessed knowledge levels (“Can you describe how GenAI works in your own words?”). The second domain explored professional involvement in GenAI adoption, including current or past engagement with artificial intelligence (AI)–related projects, training programs, or policy development. The third domain examined attitudes and decision-making processes, investigating factors influencing institutional adoption choices such as resource allocation priorities, staff readiness assessments, and perceived utility. Notably, the protocol included two interview guides for the first and second interview waves. The core components remained consistent, with modifications made to questions in the second interview guide to reflect changes over time (Multimedia Appendix 1).

The interview protocol underwent a 3-stage development process. Initial questions were derived from a systematic review of GenAI adoption literature [6-13], which were refined through pilot testing with two TCM administrators not included in the study sample. The pilot testing identified ambiguous terminology requiring simplification. Final revisions emerged from iterative discussions among the research team, involving three rounds of role-played interviews to standardize probing techniques [22-25].

ZZ, JZ, MX, and YZ conducted the interviews. All participants knew ZZ professionally. To minimize bias arising from these relationships, each interviewer was assigned only participants at or above their organizational level. This arrangement reduced potential power differentials and social desirability effects.

Interviews were recorded with consent. Audio recordings were transcribed verbatim within 48 hours of collection, with transcriptions cross-verified against original recordings by an independent researcher to ensure accuracy [26,27].

### Data Analysis

The analysis followed Colaizzi’s data analysis method [28], implemented manually through collaborative team discussions. Researchers first immersed themselves in the data by independently reading all transcripts twice, once for holistic understanding and again to identify significant statements. Through iterative discussion, the team clustered these meanings into preliminary themes, which were further condensed into core thematic categories through constant comparative analysis. The final analytic phase involved member checking, where findings were shared with 10 participants to confirm interpretive accuracy [29,30].

### Analytical Rigor

Analytical rigor was ensured through multiple verification strategies. Peer debriefing involved reviews with an external qualitative researcher who critiqued emerging themes and challenged interpretive assumptions, refining conclusions about decision-making dynamics [31]. An audit trail documented the key decisions of team discussions [32]. Member checking engaged participants in reviewing anonymized theme summaries for clarification. Reflexivity was maintained via researcher discussions on preconceptions about GenAI’s role, enabling explicit bracketing during interpretation [33]. The strategies collectively addressed credibility through external scrutiny, dependability via process transparency, confirmability by tracing analytical decisions, and transferability through contextual descriptions of institutional settings. Rigor was further strengthened by cross-verifying transcripts against recordings to ensure consistency between raw data and interpreted findings.

### Ethical Considerations

We received an ethics exemption from the Academic Ethics Committee of Hebei University of Chinese Medicine/Hebei Province Traditional Chinese Medicine Hospital according to its institutional policy, as the study involved no patients, patient data, or interventions and posed minimal risk for participants. Participants provided verbal informed consent before the first interview and verbal reaffirmation at the 6-month follow-up. We did not provide compensation to participants in this study. All data were anonymized using numeric identifiers (P1-P32, followed by a role code of H for hospital director/deputy and D for department head/deputy, and a code W1/2 for interview wave; eg, P1-HW2 means participant 1 who is a hospital director/deputy and the quote is from the second interview wave). All identifying information was removed from both the demographic data and transcriptions.

Audio files and transcripts were stored on two password-protected flash drives, with one for backup, which were kept separately by the first and the corresponding authors, along with the hard copies such as field notes and discussion meeting minutes. The data were accessible only to the research team. Participants retained the right to withdraw data until the final analysis phase [34,35].

## Results

### Participant Characteristics

A total of 32 participants were interviewed, all of whom completed both interview waves, resulting in a final dataset of 64 interviews. Interviews averaged 39.3 (range 21-47) minutes during the first wave and 44.7 (range 29-52) minutes during the second wave. A total of 32 leaders (12 women, 20 men) from 3 tertiary hospitals (1 TCM hospital in Hebei and 2 Western medicine hospitals in Sichuan and Guangdong) participated. Ages ranged from 39 to 53 (mean 46.4) years, and leadership tenure spanned 4-17 (median 11.3) years. Participants included 8 (25%) hospital directors/deputies and 24 (75%) department heads/deputies, 2 of whom were promoted to deputy director by the second interview. The cohort was a good representation of the demographic characteristics of Chinese hospital leaders (Table 1).

**Table 1.** Demographic characteristics of participants (N=32).

Characteristic	Values
Overall age (years)^a^, average (range)	46.4 (39-53)
Age by role (years), average (range)	
Hospital director/deputy	48.3 (43-53)
Department head/deputy	44.1 (39-48)
Sex, n (%)	
Female	12 (37.5)
Male	20 (62.5)
Hospital location, n (%)^b^	
Hebei	11 (34.4)
Sichuan	12 (37.5)
Guangdong	9 (28.1)
Position, n (%)^c^	
Hospital director/deputy	8 (25.0)
Department head/deputy	24 (75.0)
Years in role^d^, median (range)	11.3 (4-17)

a Age at study enrollment.

b Hebei: 1 tertiary hospital specialized in traditional Chinese medicine. Sichuan and Guangdong: 2 tertiary hospitals of Western medicine.

c Position at the time of enrollment, with 2 department heads promoted to deputy hospital directors by the second wave of interviews.

d Years in leadership role at the time of enrollment.

Our analysis of interview transcriptions revealed three main themes, as follows.

### Theme 1: Enhanced Awareness and Knowledge of GenAI

Our interviews revealed a marked improvement in participants’ understanding of GenAI over the 6-month period. Compared with the first wave of interviews, a substantially higher number of participants in the second wave demonstrated awareness and knowledge about GenAI. While only 17 participants (13 department heads/deputies, 4 hospital directors/deputies) initially were able to correctly describe GenAI in their own language and name examples of GenAI technology and possible professional scenarios for application, it increased to 29 participants in the second wave (22 department heads/deputies, 7 hospital directors/deputies). Participants attributed this enhanced understanding to a variety of knowledge sources, including media coverage, discussions with peers and staff, professional conventions, and direct involvement in GenAI projects:

AI has been such a hot topic recently. Hard not to notice it. All over the news. People talk about it too. Some of my staff members already started using it. They sometimes tell me about how useful it is. So I looked it up and started using it too.

[P1-DW2]

I didn’t really know what GenAI is and what it can be used for in the first place. Even had doubts when others told me about it...I saw it on the news. Presentations on conventions, too. Lots of discussion about it...I have taken some time to read some articles (about it).

[P21-HW2]

As a matter of fact, I wasn’t knowledgeable about AI in the first place. I learned along the way, especially after participating in the creation of a TCM AI model. The discussions with experts and firsthand exposure to the technology taught me a lot about it. Of course, I did some learning on my own. Otherwise I can’t even understand what others say about it.

[P22-HW2]

### Theme 2: Supportive Attitudes Toward GenAI Adoption in Professional Settings

#### Overview

Across both interview waves, all participants expressed supportive attitudes toward adopting GenAI technologies in professional settings. However, variances emerged when examining differences by leadership roles and over time. Many participants initially showed reluctance toward formal institutional adoption. Instead, they tended to encourage personal applications of GenAI at no cost. Notably, a shift occurred in the second interview wave, particularly among hospital directors/deputies, who began to endorse or plan for formal institutional implementation with allocated budgets.

#### Subtheme 1: Universal Supportive Attitudes

All participants, regardless of their leadership roles, expressed a baseline supportive attitude toward GenAI. Many described GenAI as an innovative tool with transformative potential for streamlining processes and enhancing efficiency. They recognized the benefits of personal experimentation and adoption of the technology and believed that its integration could eventually lead to broader institutional improvements:

Technologies such as this (GenAI) are inevitable. They can enormously enhance work efficiency. It is the future. I strongly support its applications. Even encourage my staff to experiment with it.

[P1-DW1]

Of course I support AI applications in hospital work. I’m open to new technologies and innovations such as this.

[P2-HW1]

Even more supportive than you last asked me. I have heard staff using it and their feedback is very good. I tried a couple of time. Remarkable indeed. For sure our hospital will adopt AI one way or another. The benefits can be evident in both clinical and management (settings).

[P13-HW2]

#### Subtheme 2: Varied Attitudes by Leadership Roles and Evolution Over Time

Despite universal supportive attitudes, differences were observed based on leadership roles. This was especially true in the first wave of interviews, when many hospital directors and senior administrators exhibited cautious or reluctant attitudes toward formal institutional adoption. They were more inclined to support, even encourage, personal applications of GenAI. Some participants noted concerns about resource allocation and formal budgeting as senior leaders:

At the director’s level, I support staff members to experiment with AI. However, I don’t see how it can be adopted at the hospital level.

[P3-HW1]

The timing is not mature yet. Not yet for pushing for hospital-level adoption, esp. committing hospital resources to doing so.

[P11-HW1]

In contrast, department leaders expressed enthusiasm from the beginning but lacked the authority to act. As one put it:

I have personally tried it and used it for many work tasks. Of course, I wish for hospital-level endorsement and commitment to support institutional adoption, but I’m not in a position to decide.

[P6-DW2]

However, a noticeable evolution in attitudes toward formalizing GenAI adoption emerged during the second interview wave. Some leaders who initially expressed reservations about committing institutional resources to GenAI projects gradually shifted toward actively supporting, planning, or even executing formal adoption strategies with allocated budgetary resources. For example, when asked about the changes and current status of their GenAI adoption in the second interview wave, P11 responded with evident enthusiasm:

Indeed very different from last time. I have not only purchased some pro versions of AI and used them in my own daily work but also considering integrating DeepSeek in our hospital. Already begun discussing with an AI company.

[P11-HW2]

### Theme 3: Evolving Obstacles and Catalysts Over Time

Our interviews reveal a clear changing process from early uncertainty to later enthusiasm as leaders moved past initial obstacles and embraced specific catalysts for GenAI adoption.

#### Subtheme 1: Initial Barriers

In the first interview wave, senior leaders described fundamental hurdles, such as a lack of awareness, limited technical literacy, and uncertainty about practical applications. Without hands-on experience or clear use cases, many saw no reason to commit resources.

How can I use something that I don’t even know about?

[P4-HW1]

Rarely (used GenAI). I’m not familiar with AI. I saw my assistant using it a couple of times. My daughter told me that it’s very useful and wanted to show me. I didn’t quite learn. Didn’t see why I need it.

[P7-HW1]

They also worried that formal investment carried career and financial risks.

To be frank, let alone that there is no budget at the hospital level, even if there is, at this point it’s hard to see how it (adopting AI at the institutional level) will help me advance my career...There are risks with investing in new technologies. As a leader yourself (interviewer), you understand this, too. We have to consider the benefits versus risks when deciding on an investment. Not that I don’t support (formal adoption), the timing is not mature.

[P22-HW1]

#### Subtheme 2: Change Catalysts

Between waves, three driving factors helped overcome the barriers and facilitated changes at different leadership levels. At the department leader level, department leaders’ early personal trials surfaced tangible benefits in supporting their daily tasks. This dispelled basic skepticism.

As I experimented with AI, my perspective changed. I can see examples of how AI could streamline my work and even help with decision-making...Many of my earlier concerns were gradually addressed.

[P6-DW2]

Peer success stories and cross-hospital observations created a bandwagon effect. Hearing that fellow institutions achieved workflow gains prompted even senior leaders to reexamine their stance.

Other hospitals are using AI already. I hear someone talking about how their hospital is using this and that AI and making this and that achievements. We will fall behind if we don’t do anything.

[P14-HW2]

Additionally, the launch of DeepSeek seemed to provide a focal point for institutional endorsement.

Especially after DeepSeek came out, everybody knows about DeepSeek. Some already have launched projects using DeepSeek.

[P14-HW2]

It wasn’t easy to get money for investing in this (GenAI adoption). At least you have to have some specific projects to apply for the budgets. Back then, I didn’t even know what the hospital could do with it...Much easier now, especially with DeepSeek. Everybody is promoting it. I have already launched a project using DeepSeek for supporting clinical decision-making.

[P29-HW2]

## Discussion

### Principal Findings

This longitudinal qualitative study investigated the evolving perceptions and attitudes of senior Chinese hospital leaders toward the adoption of GenAI in professional settings over a 6‐month period. Three key themes emerged: a significant improvement in the leaders’ understanding of GenAI, a generally supportive attitude toward its adoption that evolved over time and varied by leadership role, and a dynamic shift in the perceived obstacles and catalysts influencing GenAI integration. Our findings provide valuable insights into the complex process of technology adoption within the unique context of Chinese hospitals, where government policies and competitive pressures drive rapid digital transformation.

The first key finding was a marked increase in participants’ awareness and knowledge of GenAI over time. Initially, fewer than half of the participants could correctly describe the concept and potential applications of GenAI. However, by the second interview wave, this number increased significantly, indicating that as leaders were exposed to diverse sources of information, including media reports, peer discussions, professional conventions, and hands-on involvement in GenAI projects, their understanding deepened. This finding supports existing research that identifies a lack of knowledge as a major barrier to AI adoption in multiple industries [36-38]. In our study, as leaders’ awareness improved, their ability to envision practical applications and assess potential benefits increased, thereby aligning with the diffusion of innovation theory, which emphasizes that early exposure and accessible knowledge are critical to moving along the adoption curve [39]. This suggests that, in promoting GenAI adoption, hospitals and policy makers should invest in targeted educational initiatives to further bridge the knowledge gap among health care leaders.

The second major theme was the supportive attitude toward GenAI adoption that was expressed across all participants, with notable evolution regarding formal institutional adoption. In the first wave, many senior leaders, particularly those in higher administrative roles, favored individual experimentation with GenAI rather than committing significant institutional resources. This cautious approach is commonly seen in early technology adoption stages, where concerns about budget allocation, uncertain return on investment, and the risks inherent in new technologies are often prevalent. By the second wave, however, hospital directors and deputies exhibited increased willingness to move beyond individual use and actively plan for institutional implementation, including formal budgeting and strategic integration. This transition reflects a growing confidence in the technology’s potential, a progression that echoes patterns observed in health care technology adoption literature, where initial caution gradually gives way to institutional endorsement as practical benefits become clearer [40]. Our findings suggest that showcasing successful pilot projects and clear return on investment pathways could accelerate the formal adoption process.

The third theme highlighted the evolving landscape of obstacles and drivers influencing GenAI adoption. In the initial phase, obstacles such as limited technical literacy, uncertainty about practical applications, skepticism regarding benefits, and a lack of dedicated budgets were predominant. These challenges resonate with previous studies that identify similar barriers in AI integration within health care [41,42]. However, as the study progressed, many of these obstacles diminished, likely as a result of improved knowledge, increased hands-on experience with GenAI, and changes in the policy and technological environment, and new drivers emerged. For instance, peer pressure from other hospitals that had begun integrating GenAI, a clearer vision of its potential applications, and the increasing availability of budgetary resources all served as catalysts. Notably, our study period coincided with DeepSeek’s launch in China (July 2024) and its global debut (August 2024). Although participants did not discuss the driving impact of DeepSeek explicitly, several leaders noted that its technical breakthrough accelerated both awareness and institutional interest in GenAI, which reinforces the notion that breakthroughs in technology can rapidly shift the adoption landscape [43,44]. Furthermore, institutional isomorphic pressures may help explain why hospital leaders converged on similar GenAI strategies [45,46], especially in the context of the high-profile debut of DeepSeek. Under coercive pressures, national policy endorsements and regulatory expectations compelled leaders to align with government-backed technology agendas. Simultaneously, normative pressures emerged as peer institutions shared GenAI successes at professional forums, creating industry norms that legitimized investment [42,47]. The dual forces reduced uncertainty, reinforced trust in GenAI’s value, and accelerated formal adoption across hospitals, demonstrating how external mandates and interorganizational learning jointly shape technology uptake. The observations align with the broader literature on technology diffusion, which posits that overcoming initial barriers through positive experiences and external validation can lead to accelerated adoption [43-45]. Leveraging coercive and normative pressures can normalize innovation, streamline budget approvals, and overcome resistance rooted in perceived risk [46,47]. Therefore, fostering a culture of knowledge and experience sharing, networking to share and showcase peer hospital GenAI implementations, and aligning institutional plans with emerging mandates can be strategies to leverage peer influence and emerging technological advancements.

Notably, the three themes are closely intertwined. Improved understanding of GenAI appears to have played a crucial role in transforming supportive attitudes toward formal adoption, as increased knowledge alleviated initial uncertainties and facilitated resource commitment. Concurrently, the mitigation of early obstacles allowed emerging catalysts, such as the influence of DeepSeek and competitive peer adoption, to drive a shift from cautious exploration to proactive institutional integration. In comparison with existing literature, our findings extend traditional technology adoption models by emphasizing the critical role of longitudinal exposure and experiential learning in overcoming barriers and activating drivers. These insights suggest that hospitals should consider implementing ongoing education and pilot initiatives, while policy makers should create frameworks that support early experimentation and gradual scaling of GenAI applications in health care.

### Implications for Practice

Our findings offer several important implications for hospital administrators and policy makers seeking to facilitate GenAI adoption. First, there is a clear need for ongoing educational initiatives aimed at senior hospital leaders to enhance their understanding of GenAI’s capabilities, limitations, and potential applications within health care. These initiatives should leverage various channels, including media, peer-to-peer learning, and professional conventions. Second, demonstrating the practical value and return on investment of GenAI through well-defined pilot projects is crucial to encourage formal institutional adoption, particularly among budget-conscious leaders. Showcasing successful use cases and addressing concerns related to resource allocation can build confidence and pave the way for broader implementation. Third, fostering a culture of innovation and knowledge sharing among hospitals can leverage the power of peer influence and accelerate the adoption process. Creating platforms for hospitals to share their experiences and best practices with GenAI implementation can be highly beneficial. Finally, continuous evaluation and strategic consideration of emerging AI technologies, such as DeepSeek, are essential to capitalize on advancements that offer improved accessibility, efficiency, and cost-effectiveness. Addressing remaining obstacles such as data privacy and ethical considerations through clear guidelines and robust security measures will also be critical for building trust and ensuring the responsible integration of GenAI in Chinese hospitals. Additionally, creating interhospital networks, showcasing peer GenAI implementations, and then integrating the insights into institutional planning in accordance with emerging policy mandates can be a potential strategy to combine normative peer influence with coercive endorsement, helping leaders normalize GenAI use, justify budget requests, and accelerate formal adoption.

### Limitations

This study has several limitations. First, our sample was drawn from three tertiary hospitals. This limited the diversity of sampling, excluding other types of hospitals in China. Second, data collection relied on telephone interviews, which could limit the depth of information compared to face-to-face interactions. Future research should consider incorporating a larger, more diverse sample and a quantitative or mixed methods design to validate and extend our findings and provide a further understanding of GenAI adoption dynamics in health care settings. Furthermore, we did not collect direct evidence to assess how DeepSeek’s launch and national-level endorsement drove GenAI uptake, which was a likely driver of participants’ changes in perception and attitudes over the study period. Future research should investigate the operational impacts of major technology rollouts, such as DeepSeek’s debut, and accompanying policy endorsements on adoption dynamics in hospital settings.

### Conclusions

Senior Chinese hospital leaders’ perceptions and attitudes toward GenAI have evolved significantly over time. Improved knowledge through diverse information channels has enhanced their comprehension and shifted their stance from initial reluctance toward support for formal institutional adoption. The dynamic interplay between diminishing obstacles and emerging catalysts, notably the influence of peer practices and technological breakthroughs such as DeepSeek, underscores the potential for transformative change in health care management. Targeted educational initiatives, pilot projects, and robust policy frameworks are needed to facilitate GenAI integration.

## Supplementary material

10.2196/75531 Multimedia Appendix 1 Interview guides (English translation).

10.2196/75531 Multimedia Appendix 2 Artificial intelligence prompts for study design support and interview guide drafting.

## Abbreviations

AI: artificial intelligence
GenAI: generative artificial intelligence
TCM: traditional Chinese medicine
