# Supplementary material for: Evolving Perceptions and Attitudes to Adopting Generative AI in Professional Settings: Multicenter Longitudinal Qualitative Study of Senior Chinese Hospital Leaders
Source: J Med Internet Res. 2025 Jun 27;27:e75531. doi: 10.2196/75531 (PMC12227188; doi:10.2196/75531)
Supplement: Multimedia Appendix 1 [file jmir-v27-e75531-s001.pdf]

Appendix 1: Interview Guide (English Translation)

1st Wave Interview Guide

1. Introduction and Consent

- Welcome participant and thank them for their time.
- Explain study purpose.
- Confirm voluntary participation, confidentiality, and audio recording consent.

2. Section 1: General Awareness of GenAI

- "Have you heard of generative artificial intelligence (GenAI)?"
  - Probe: "Which sources informed you about it?"
- "Can you describe how GenAI works in your own words?"
  - Probe: "What features or functions stand out to you?"
- "How would you rate your current knowledge of GenAI?"
  - Probe: "What factors influence that rating?"

3. Section 2: Professional Involvement

- "Have you participated in any AI-related projects or training?"
  - Probe: "What was your specific role?"
- "Can you share examples of policies or guidelines your institution has developed for AI?"
  - Probe: "Who led the efforts?"
- "What challenges did you encounter when engaging with GenAI?"
  - Probe: "How were those challenges addressed?"

4. Section 3: Attitudes and Decision Factors

- "What factors would influence your decision to adopt GenAI at your hospital?"
  - Probe: "Consider cost, staff readiness, and patient impact."
- "How do you perceive risks related to adopting GenAI in your professional practice?"
  - Probe: "What safeguards would you require?"
- "What potential benefits do you foresee from using GenAI?"

- Probe: “Which clinical or administrative areas might gain most?”

5. Closing

- Invite additional comments or concerns.
- Reconfirm interest in the second-wave interview and make an appointment.

Thank participant and end recording.

2nd Wave Interview Guide

1. Re-Introduction and Consent

- Welcome participant and thank them for returning.
- Remind them of study purpose and six-month span.
- Reconfirm voluntary participation, confidentiality, and audio recording consent.

2. Section 1: Evolving Awareness and Knowledge

- "Since our last interview, how has your awareness of GenAI changed?"
  - Probe: "Which new information sources influenced you?"
- "Can you now describe GenAI's core functions more fully?"
  - Probe: "What aspects seem most critical today?"
- "How would you rate your current knowledge compared to six months ago?"
  - Probe: "What experiences drove that change?"

3. Section 2: Updated Professional Engagement

- "What GenAI-related projects or training have you undertaken since August?"
  - Probe: "What role did you play, and what outcomes emerged?"
- "Have any new policies or guidelines been developed at your institution?"
  - Probe: "Who led these efforts and why?"
- "What operational or logistical challenges have arisen during implementation?"
  - Probe: "How did you and your team address those hurdles?"

4. Section 3: Shifting Attitudes and Decision Drivers

- "How have your views on the benefits of GenAI evolved?"
  - Probe: "Which applications have proven most valuable?"
- "Have your concerns around risks (privacy, security, ethics) changed?"
  - Probe: "What new safeguards or governance measures are now in place?"
- "What factors now most strongly influence your adoption decisions?"
  - Probe: "Consider budget reallocations, staff readiness, and patient

feedback."

5. Closing

- Invite any additional reflections or concerns.

Thank participant and end recording.
