# Supplementary material for: Evolving Perceptions and Attitudes to Adopting Generative AI in Professional Settings: Multicenter Longitudinal Qualitative Study of Senior Chinese Hospital Leaders
Source: J Med Internet Res. 2025 Jun 27;27:e75531. doi: 10.2196/75531 (PMC12227188; doi:10.2196/75531)
Supplement: Multimedia Appendix 2 [file jmir-v27-e75531-s002.pdf]

Prompt used for study design

我们做了一个研究设计，请你仔细看看。我们现在遇到一个问题，不确定应该选择描述性质性研究还是现象学执行研究，你觉得哪一个更适合我们这个研究？具体说明为什么？

We have developed a study design—please review it carefully. We now face a question: we're not sure whether to choose descriptive qualitative research or a phenomenological approach. Which do you think is more suitable for our study? Please explain your reasoning in detail.

Prompts used in interview guide drafting

请帮我们润色一下这个访谈指南的语言。注意，不要改变里面问题的意思，也不要改变整体结构，只润色语言。

Please help us polish the language of this interview guide. Do not change the meaning of any questions or alter the overall structure—just refine the wording.

请根据上传的这个访谈指南，拟一个用于六个月之后再次对同一组访谈对象进行访谈的访谈指南。注意保持核心问题一致，整体结构一致，只是在问题措辞和背景上体现出"经过六个月之后"这个上下文。

Based on the uploaded interview guide, draft a version for interviewing the same participants six months later. Keep the core questions consistent and the structure the same, but adjust the phrasing and context to reflect that it is taking place 'after six months.'
